# Readmission Risk Factors and Complications in Stevens-Johnson Syndrome and Toxic Epidermal Necrolysis

**DOI:** 10.7759/cureus.7631

**Published:** 2020-04-11

**Authors:** Yiran Jiang, Tyler Sharpe

**Affiliations:** 1 Internal Medicine, University of Louisville School of Medicine, Louisville, USA; 2 Internal Medicine, University of Louisville, Louisville, USA

**Keywords:** stevens-johnson syndrome, toxic epidermal necrolysis, readmission, post-hospitalization, complications, cmv

## Abstract

Stevens-Johnson syndrome (SJS) and toxic epidermal necrolysis (TEN) are mucocutaneous hypersensitivity reactions that cause necrosis of the epidermis, often at multiple sites. This process is frequently caused by medications and is associated with significant complications and mortality during hospitalization. However, increasing attention has been drawn to the less studied area of post-hospitalization complications leading to multiple readmissions. We present a patient diagnosed with trimethoprim-sulfamethoxazole induced SJS/TEN, who was readmitted within one week with sepsis. We also discuss the readmission risk factors and post-hospitalization features and complications of SJS/TEN.

## Introduction

Stevens-Johnson syndrome (SJS) is named after pediatricians Albert Stevens and Frank Johnson, who published a case report of two patients in 1922 [1]. SJS and toxic epidermal necrolysis (TEN) are type IV delayed hypersensitivity reactions that form two poles of a spectrum of disease. The difference is defined by the degree of skin involvement: SJS if less than 10% of total body surface area involved, SJS/TEN overlap if between 10 to 30% skin is involved, and TEN if greater than 30% of skin involvement [2, 3]. Medications are the most common cause, but infections, both bacterial and viral, have also been identified as causes. SJS/TEN has become a recognized disease process that constitutes a medical emergency, with early diagnosis and management being critical to survival. Just as important, but less well recognized, are peridischarge complications. Due to the often-critical state of hospitalized patients with these diseases, they are at significantly increased risk for readmission for a variety of reasons. Here, we present a patient who was readmitted less than one week after discharge following SJS/TEN overlap due to trimethoprim-sulfamethoxazole. We also discuss the risk factors for readmission, as well as common post-hospitalization complications.

## Case presentation

A 71-year-old African American female with a past medical history of chronic obstructive pulmonary disease, type 2 diabetes mellitus, and hypertension presented from a nursing facility due to increasing lethargy of one-day duration. She was discharged six days prior after hospitalization for SJS/TEN overlap secondary to trimethoprim-sulfamethoxazole. During that hospitalization, she completed etanercept as well as a steroid course, with the improvement of skin breakdown prior to discharge. On the initial presentation, patient vitals were significant for the blood pressure of 97/37, a heart rate of 101. Physical exam was significant for mild confusion and diffused patchy erosions and skin sloughing present on both upper and lower extremities, inguinal area, buttocks, and over the scalp - improved from prior discharge. Her initial metabolic panel was significant for an albumin of 1.4 g/dL and creatinine of 1.76 mg/dL (baseline <1 mg/dL). Her complete blood count (CBC) was unremarkable. Head CT did not show acute abnormalities.

She was admitted to the intensive care unit (ICU) with acute encephalopathy and hypotension, which initially improved with fluids, vancomycin, and piperacillin/tazobactam. On day 3, she was transferred to the floor but soon developed a fever of 102 F, tachycardia to 120, and tachypnea to 20. The suspected source at this time was skin and soft tissue infection, as she had some mild erythema and tenderness to palpation overlying an area of skin breakdown. Due to ongoing fever, tachycardia, and leukocytosis despite empiric intravenous (IV) antibiotics, CT chest and abdomen pelvis were ordered, with the result unrevealing for a source of infection. An MRI of the pelvis was negative for an abscess or osteomyelitis. The autoimmune workup was unremarkable. A transesophageal echocardiogram was negative for vegetations. She continued to have fevers despite broad-spectrum antibiotics.

**Figure 1:**
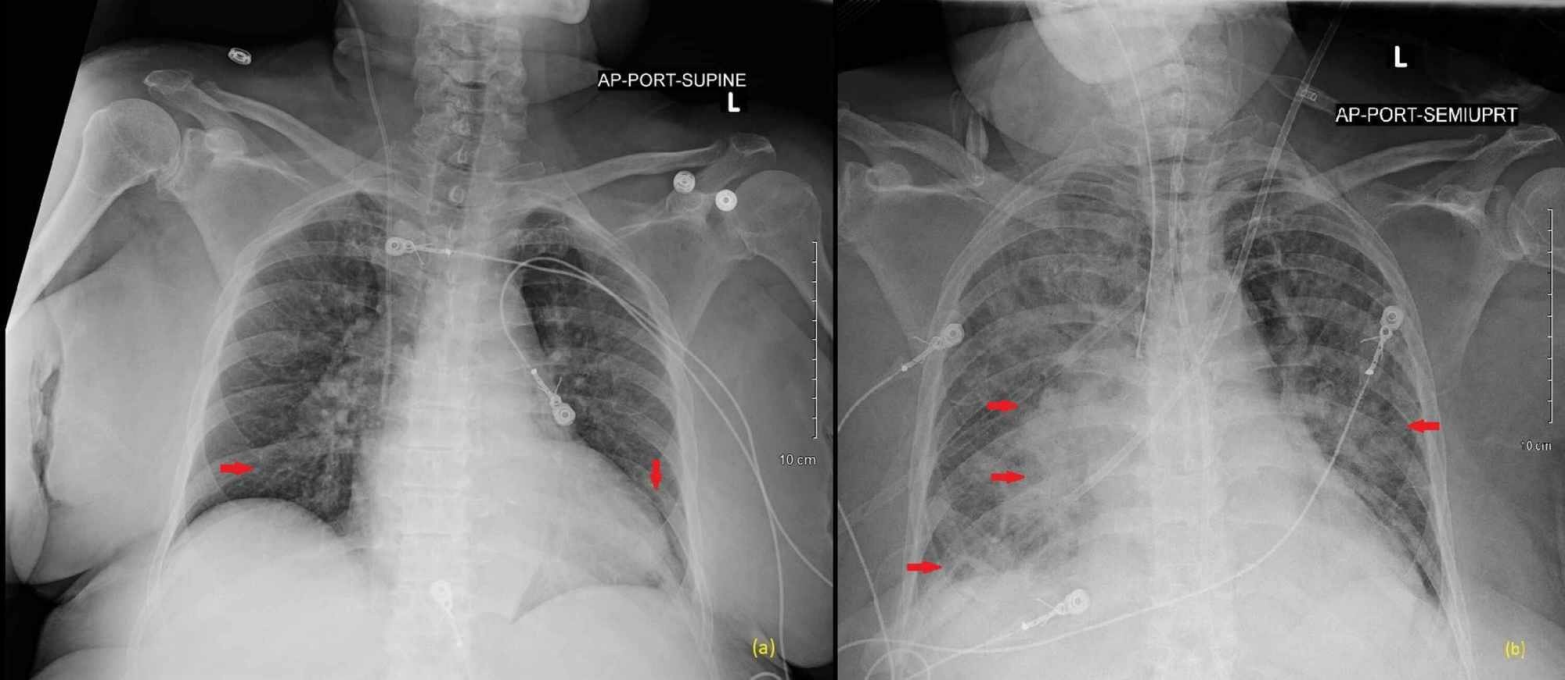
Portable chest radiograph (a) X-ray on admission showing low lung volumes and bibasilar atelectasis; (b) follow up X-ray on day 15 showing diffuse bilateral multifocal opacities greater in the right lung consistent with pneumonia.

Further infectious workup revealed cytomegalovirus (CMV) immunoglobulin M (IgM) positivity with a quantitative polymerase chain reaction (PCR) of 5740 units/mL. CMV IgG and Epstein-Barr virus (EBV) immunoglobulin G (IgG) were also positive. EBV IgM and herpes simplex virus (HSV) were negative. Sacral wound swabs also returned positive for *Candida albicans*. On day 11 of admission, the patient's antibiotic regimen was switched to valganciclovir and fluconazole. Her subsequent hospital stay was further complicated by acute respiratory failure requiring intubation for four days (Figure 1). A bronchoscopy was performed, and the results were consistent with CMV pneumonitis. The patient ultimately completed a 10-day course of valganciclovir for a diagnosis of CMV viremia and was monitored off of antimicrobials for recovery. She was discharged to the rehabilitation after a 28-day hospital stay.

## Discussion

SJS/TEN is a rare (estimated incidences of two to seven cases per million per year), but potentially life-threatening disease with one-year mortality reaching up to 34% [4, 5]. Often, epidemiological, medical, and social factors that lead to readmission after initial diagnosis are overlooked.

In terms of the epidemiology of patients at risk for readmission, a review of the literature shows many contributing factors. Broadly speaking, age and comorbidities are significant reasons. A recent study looking at predictors of 30-day readmission noted an increase in rates for patients over the age of 45, with no preference for sex. Comorbidities most significantly associated with readmission include diabetes with complications, HIV/AIDS, and malignancies [5, 6]. Increased risk of HIV/AIDS and malignancies may have to do with the cessation of offending medications and polypharmacy, respectively. Patients with HIV/AIDS exemplify a population in which the cessation of a medication causing SJS/TEN should prompt a timely evaluation for an alternative as a delay in treatment can lead to increased morbidity and mortality. Although our patient did not have HIV, her causative antibiotic trimethoprim-sulfamethoxazole is often used as a prophylaxis for pneumocystis pneumonia for HIV patients. In a similar vein, patients undergoing chemotherapy often require exposure to multiple high-risk medications simultaneously, and the removal of any offending medications necessitates an evaluation for an alternative. 

Post-hospitalization complications that lead to readmission most frequently include sepsis and recurrent inflammatory skin conditions. In the short term, systemic infections and inflammatory skin conditions accounted for nearly half of readmissions - 22.0% and 20.6%, respectively [5]. This is further supported by additional research looking at extended timelines for up to three months [6]. Systemic infections are of particular concern as the combination of damaged skin continuity, and immunosuppressive treatments place these patients in a high-risk group. While microorganisms such as *Staphylococcus aureus* and *Pseudomonas aeruginosa *are of great import in this population, viral infections should also be considered. Of relevance to our patient, it is not clear whether severe illness and immunosuppressing medications lead to a reactivation of certain viruses or a novel infection. There were no characteristic CMV cutaneous findings which are more clinically consistent with reactivation of latent infection, and CMV reactivations are better documented in Drug Reaction with Eosinophilia and Systemic Symptoms (DRESS) as opposed to SJS/TEN [7]. Nevertheless, viral etiologies should remain a consideration, especially in patients who do not respond as expected to antibiotics.

One population consistently was shown to have worse outcomes are underinsured and low-income patients. The reasons for this are complex and not limited only to SJS/TEN. Social factors such as disability, housing, and poverty have been independently linked to hospital readmissions [8]. While some of these factors are admittedly difficult to change in an inpatient setting, one aspect that can be arranged is peridischarge follow up. Highly specific to SJS/TEN, inpatient dermatology consultation and subsequent dermatology follow up has been shown to lower odds of readmission by up to 10-fold [9]. In the setting of critical diseases such as SJS/TEN, awareness of the barriers for underinsured and low-income patients, as well as elderly patients, are essential to a successful post-hospitalization course.

## Conclusions

SJS/TEN is a devastating disease process which poses both an immediate and sustained challenge to survival and quality of life. Often overlooked in an inpatient setting are factors and considerations that may lead to readmissions, further impacting a patient's quality of life. A recognition of the epidemiologic, medical, and social factors that interplay in a patient’s peridischarge and post-hospitalization period is a crucial first step to improving the long-term outcomes for these patients.

## References

[1] Stevens AM, Johnson FC (1922). A new eruptive fever associated with stomatitis and ophthalmia. Am J Dis Child.

[2] Ishida T, Kano Y, Mizukawa Y, Shiohara T (2014). The dynamics of herpesvirus reactivations during and after severe drug eruptions: their relation to the clinical phenotype and therapeutic outcome. Allergy.

[3] Bastuji-Garin S, Rzany B, Stern RS, Shear NH, Naldi L, Roujeau J (1993). Clinical classification of cases of toxic epidermal necrolysis, stevens-johnson syndrome, and erythema multiforme. Arch Dermatol.

[4] Rzany B, Mockenhaupt M, Baur S (1996). Epidemiology of erythema exsudativum multiforme majus, Stevens-Johnson syndrome, and toxic epidermal necrolysis in Germany ( 1990-1992): structure and results of a population-based registry. J Clin Epidemiol.

[5] Guzman AK, Zhang M, Kwatra SG, Kaffenberger BH (2020). Predictors of 30-day readmission in Stevens-Johnson syndrome and toxic epidermal necrolysis: a cross-sectional database study. J Am Acad Dermatol.

[6] Cheng BT, Silverberg JI (2020). Long‐term and multiple hospital readmissions after discharge for Stevens-Johnson syndrome and toxic epidermal necrolysis. Br J Dermatol.

[7] Kano Y, Hiraharas K, Sakuma K, Shiohara T (2006). Several herpesviruses can reactivate in a severe drug‐induced multiorgan reaction in the same sequential order as in graft‐versus‐host disease. Br J Dermatol.

[8] Joynt Maddox KE, Reidhead M, Jianhui H (2019). Adjusting for social risk factors impacts performance and penalties in the hospital readmissions reduction program. Health Serv Res.

[9] Milani-Nejad N, Zhang M, Kaffenberger BH (2017). Association of dermatology consultations with patient care outcomes in hospitalized patients with inflammatory skin diseases. JAMA Dermatol.

